# Mortality of major cardiovascular emergencies among patients admitted to hospitals on weekends as compared with weekdays in Taiwan

**DOI:** 10.1186/s12913-021-06553-7

**Published:** 2021-05-29

**Authors:** Chao-Lun Lai, Raymond Nien-Chen Kuo, Ting-Chuan Wang, K. Arnold Chan

**Affiliations:** 1 Department of Internal Medicine, National Taiwan University Hospital Hsin-Chu Branch, Hsin-Chu, Taiwan; 2 Department of Internal Medicine, College of Medicine, National Taiwan University, Taipei, Taiwan; 3 Institute of Epidemiology and Preventive Medicine, College of Public Health, National Taiwan University, Taipei, Taiwan; 4 Institute of Health Policy and Management, College of Public Health, National Taiwan University, Taipei, Taiwan; 5 Health Data Research Center, National Taiwan University, Taipei, Taiwan; 6 Graduate Institute of Oncology, College of Medicine, National Taiwan University, Taipei, Taiwan

**Keywords:** Mortality; weekend; weekday, Cardiovascular emergency, Aortic aneurysm, Myocardial infarction, Ischemic stroke, Pulmonary embolism

## Abstract

**Background:**

Several studies have found a so-called weekend effect that patients admitted at the weekends had worse clinical outcomes than patients admitted at the weekdays. We performed this retrospective cohort study to explore the weekend effect in four major cardiovascular emergencies in Taiwan.

**Methods:**

The Taiwan National Health Insurance (NHI) claims database between 2005 and 2015 was used. We extracted 3811 incident cases of ruptured aortic aneurysm, 184,769 incident cases of acute myocardial infarction, 492,127 incident cases of ischemic stroke, and 15,033 incident cases of pulmonary embolism from 9,529,049 patients having at least one record of hospitalization in the NHI claims database within 2006 ~ 2014. Patients were classified as weekends or weekdays admission groups. Dates of in-hospital mortality and one-year mortality were obtained from the Taiwan National Death Registry.

**Results:**

We found no difference in in-hospital mortality between weekend group and weekday group in patients with ruptured aortic aneurysm (45.4% vs 45.3%, adjusted odds ratio [OR] 1.01, 95% confidence interval [CI] 0.87–1.17, *p* = 0.93), patients with acute myocardial infarction (15.8% vs 16.2%, adjusted OR 0.98, 95% CI 0.95–1.00, *p* = 0.10), patients with ischemic stroke (4.1% vs 4.2%, adjusted OR 0.99, 95% CI 0.96–1.03, *p* = 0.71), and patients with pulmonary embolism (14.6% vs 14.6%, adjusted OR 1.02, 95% CI 0.92–1.15, *p* = 0.66). The results remained for 1 year in all the four major cardiovascular emergencies.

**Conclusions:**

We found no difference in either short-term or long-term mortality between patients admitted on weekends and patients admitted on weekdays in four major cardiovascular emergencies in Taiwan.

**Supplementary Information:**

The online version contains supplementary material available at 10.1186/s12913-021-06553-7.

## Introduction

The level of staffing in hospitals is often lower on weekends than on weekdays. Decreased staffing is associated with adverse outcomes in intensive care unit patients and increases in hospital workload are associated with increases in medical adverse events [1, 2]. In 2001, Bell and Redelmeier reported that patients with serious medical conditions were more likely to die in the hospital if they were admitted on a weekend than if they were admitted on a weekday [3]. Many studies concerning the concept of a so-called weekend effect on patient mortality rates have been published thereafter and most of those studies concluded that outcomes for patients admitted at the weekends were worse despite high levels of heterogeneity [4, 5]. We conducted this retrospective study to explore the weekend effect in four major cardiovascular emergencies, i.e. ruptured aortic aneurysm, acute myocardial infarction, ischemic stroke, and pulmonary embolism, using a nationwide health insurance database in Taiwan.

## Methods

### Data sources

Since 1995, Taiwan has provided a compulsory, universal National Health Insurance (NHI) coverage for all its citizens. Patient identification number, gender, birthdate, date of outpatient clinic visit, date of hospital admission and discharge, diagnoses, procedures administered, date of pharmacy dispensing and drugs dispensed are available in the NHI claims database. The diagnoses are coded according to the International Classification of Diseases Ninth Revision Clinical Modification (ICD-9-CM) system. The diagnosis codes for ischemic stroke [6, 7], and acute myocardial infarction [8] in the Taiwan NHI claims database have been validated. The Taiwan NHI claims database links to the Taiwan National Death Registry, and exact date of death can be obtained using a patient’s identification number. To comply with Taiwanese privacy regulations, all personal identifiers were encrypted and all data were analyzed anonymously. The study protocol was reviewed and exempted from approval by the Institutional Review Board of the National Taiwan University Hospital Hsin-Chu Branch.

### Study design and cohort definition

**Fig. 1:**
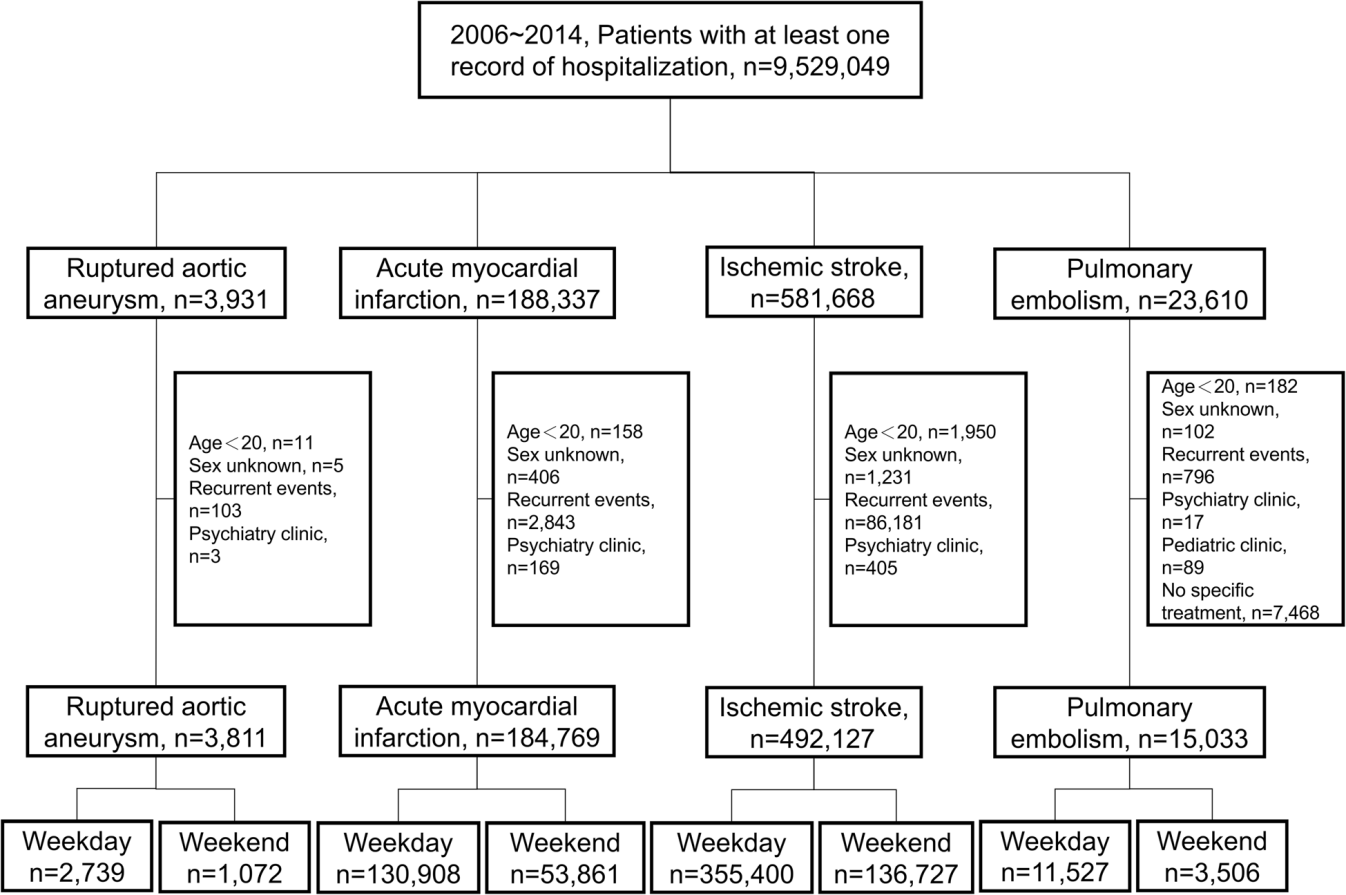
Patient flow diagram

We used the NHI claims database between 2005 and 2015 with a retrospective cohort study design. All adult beneficiaries aged ≥20 years with at least one record of hospitalization within the Jan. 01 2006 ~ Dec. 31 2014 enrollment period were identified. Patients with diagnoses of ruptured aortic aneurysm (ICD-9-CM: 441.1, 441.3, 441.5, 441.6), acute myocardial infarction (ICD-9-CM: 410.x0, 410.x1), ischemic stroke (ICD-9-CM: 433.x1, 434.x1, 435.9, 436, 437.1x, 437.9x) [9, 10] and pulmonary embolism (ICD-9-CM: 415.1, 673.2, v12.51) were further extracted to create four study subsets. For patients admitted to one hospital and then transferred to another, only one admission was considered and only the first event for each patient within each subset was retained for analysis. Patients with unknown gender or hospitalization in psychiatry clinics were excluded. For pulmonary embolism subset, we also excluded patients with hospitalization in pediatric clinics and patients who did not receive any specific treatments for pulmonary embolism such as anticoagulant therapy, fibrinolytic therapy, surgical embolectomy, and trans-catheter embolectomy (Fig. 1).

### Definitions of weekend/weekday and follow-up

We defined weekend group as patients who were admitted to hospital on Saturday, Sunday and national festival days in Taiwan within the 2006 ~ 2014 enrollment period. All other times were defined as weekdays. The date of the admission of the index hospitalization was defined as the “index date.” All patients were followed until death or 365 days after discharge from the index hospitalization whichever came first. The clinical outcomes included in-hospital mortality and one-year mortality. Exact dates of death were obtained from the Taiwan National Death Registry.

### Background characteristics

We identified characteristics about the first hospital visited by each patient such as hospital level, teaching or non-teaching hospital, public or private hospital, number of acute beds, number of physician specialists, and volume of specific procedures for each specific disease within a one-year period prior to index date. Age and gender of the attending physician who carried out the specific procedures for each patient were also identified. As for characteristics of patients, age, gender, Taiwan NHI premium, year of onset, total number of outpatient clinic visits and total number of hospitalizations within the one-year period prior to index date were also identified. Comorbidities were evaluated by Elixhauser’s comorbidities and defined as the appearance of one or more of the specific diagnosis codes twice in the outpatient records or once in the inpatient records within the one-year period prior to the index date and then coded as binary variables [11]. Any medications administered were extracted from the NHI claims database within the one-year period prior to the index date [12]. Only comorbidities and medications with a prevalence of more than 1.0% were retained in the analysis. Finally, whether the patient had received hospital transfer or not and specific procedures/therapies the patient had received were identified.

### Statistical analysis

Continuous variables are presented as mean (standard deviation) and categorical data are presented in contingency tables. We used standardized difference to measure covariate balance between weekend and weekday groups among each study subset, whereby an absolute standardized difference of greater than 0.10 represented meaningful imbalance. A logistic regression model was used for estimation of the relative risks (odds ratios [ORs]) of various clinical outcomes in the weekend group compared with the weekday group with adjustment of all the background characteristics mentioned above. We also performed pairwise comparison between patients admitted on different weekdays using the same logistic regression model. All analysis was performed using SAS software, version 9.4 (SAS Institute, Inc., Cary, North Carolina).

## Results

### Characteristics of patients in the study population

Through 2006 to 2014, we identified 9,529,049 patients having at least one record of hospitalization in the NHI database. Of them, we identified and extracted 3811 incident cases of ruptured aortic aneurysm (2739 were admitted on a weekday, 1072 admitted on a weekend), 184,769 incident cases of acute myocardial infarction (130,908 were admitted on a weekday, 53,861 admitted on a weekend), 492,127 incident cases of ischemic stroke (355,400 were admitted on a weekday, 136,727 admitted on a weekend), and 15,033 incident cases of pulmonary embolism (11,527 were admitted on a weekday, 3506 admitted on a weekend) (Fig. 1). Compared with the weekday group, patients admitted at the weekend were less likely to be presented to district hospitals, to be presented in 2009, and to have prior hospitalization within one-year period prior to index date in the ruptured aortic aneurysm subset (Supplementary Table 1). We found no difference in all the comparisons of background characteristics between the weekend group and the weekday group in the acute myocardial infarction, ischemic stroke, and pulmonary embolism study subsets (Supplementary Table 2, Supplementary Table 3 and Supplementary Table 4).

### Clinical outcomes

Among the ruptured aortic aneurysm subset, the in-hospital mortality was 45.3% in the weekday group and 45.4% in the weekend group (standardized difference − 0.0017) while the one-year mortality was 62.2% in the weekday group and 62.7% in the weekend group (standardized difference − 0.0090), respectively (Supplementary Table 1). Among the acute myocardial infarction subset, the in-hospital mortality was 16.2% in the weekday group and 15.8% in the weekend group (standardized difference 0.0118), while the one-year mortality was 30.9% in the weekday group and 30.2% in the weekend group (standardized difference 0.0164), respectively (Supplementary Table 2). Among the ischemic stroke subset, the in-hospital mortality was 4.2% in the weekday group and 4.1% in the weekend group (standardized difference 0.0044), while the one-year mortality was 16.2% in the weekday group and 15.6% in the weekend group (standardized difference 0.0145), respectively (Supplementary Table 3). Among the pulmonary embolism subset, the in-hospital mortality was 14.6% in the weekday group and 14.6% in the weekend group (standardized difference − 0.0009), while the one-year mortality was 35.4% in the weekday group and 35.4% in the weekend group (standardized difference − 0.0003), respectively (Supplementary Table 4).

### Regression analysis

**Fig. 2:**
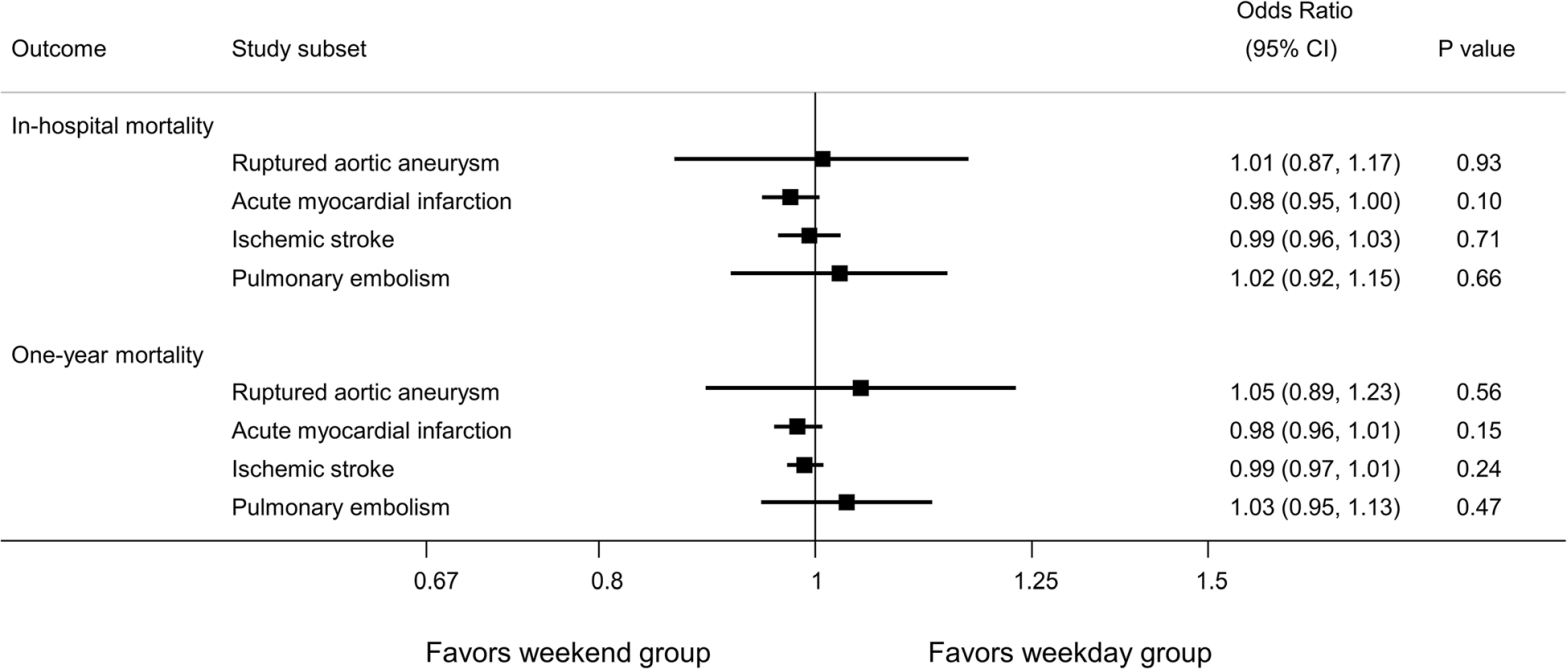
Summary of relative risks of various clinical outcomes in weekend group compared with weekday group stratified by study subsets. Abbreviation: CI, confidence interval

Among the ruptured aortic aneurysm subset, we found no difference in risk of in-hospital mortality (adjusted OR 1.01, 95% confidence interval [CI] 0.87–1.17, *p* = 0.93) and risk of one-year mortality (adjusted OR 1.05, 95% CI 0.89–1.23, *p* = 0.56) in the weekend group compared with the weekday group. Among the acute myocardial infarction subset, we found no difference in risk of in-hospital mortality (adjusted OR 0.98, 95% CI 0.95–1.00, *p* = 0.10) and risk of one-year mortality (adjusted OR 0.98, 95% CI 0.96–1.01, *p* = 0.15) in the weekend group compared with the weekday group. Among the ischemic stroke subset, we found no difference in risk of in-hospital mortality (adjusted OR 0.99, 95% CI 0.96–1.03, *p* = 0.71) and risk of one-year mortality (adjusted OR 0.99, 95% CI 0.97–1.01, *p* = 0.24) in the weekend group compared with the weekday group. Among the pulmonary embolism subset, we found no difference in risk of in-hospital mortality (adjusted OR 1.02, 95% CI 0.92–1.15, *p* = 0.66) and risk of one-year mortality (adjusted OR 1.03, 95% CI 0.95–1.13, *p* = 0.47) in the weekend group compared with the weekday group (Fig. 2).

As for the pairwise comparison between different weekday groups, the relative risks of in-hospital mortality and one-year mortality were numerically equal between groups and most were statistically insignificant (Supplementary Table 5, Supplementary Table 6, Supplementary Table 7, and Supplementary Table 8).

## Discussion

In this large retrospective cohort analysis including 695,740 patients extracted from a total of 9,529,049 patients with records of hospitalization, we explored the weekend effect on short-term and long-term mortality of four different cardiovascular emergencies, i.e. ruptured aortic aneurysm, acute myocardial infarction, ischemic stroke, and pulmonary embolism, using the nationwide insurance database between 2005 and 2015 in Taiwan. We found no difference in either in-hospital mortality or one-year mortality between patients admitted at the weekends and patients admitted on weekdays in all the four different cardiovascular emergencies.

As early as 1978, MacFarlane had reported that perinatal mortality was higher among babies born at weekends than among those born on weekdays in England and Wales [13]. In 2001, Bell and Redelmeier explored six different medical emergencies including ruptured abdominal aortic aneurysm, acute epiglottitis, pulmonary embolism, acute myocardial infarction, intracerebral hemorrhage, and acute hip fracture in Canada. They found patients with diagnoses of ruptured abdominal aortic aneurysm, acute epiglottitis, and pulmonary embolism were more likely to die in the hospital if they were admitted on a weekend than if they were admitted on a weekday [3]. However, the weekend effect did not exist in patients with diagnoses of acute myocardial infarction, intracerebral hemorrhage, and acute hip fracture [3]. Many studies concerning the concept of a weekend effect on a patient’s survivability have been published thereafter with high levels of heterogeneity in study designs and conclusions. Furthermore, meta-analyses of a large number of studies have concluded that hospital inpatients admitted during weekends may have a higher mortality rate compared with inpatients admitted during the weekdays [4, 5].

Ruptured aortic aneurysm represents a major life-threatening condition that is associated with high mortality rates even in centers with advanced technology and high levels of expertise in cardiovascular surgery. In addition to the report conducted in Canada [3], studies conducted in Italy [14] and the United States [15] both concluded that weekend admission for ruptured aortic aneurysm was associated with an increased mortality when compared to those admitted on weekdays. Although Dasari and colleagues found more delay in reperfusion time and higher in-hospital mortality in patients presenting during off-hours than patients presenting during on-hours using the Acute Coronary Treatment and Intervention Outcomes Network-Get With The Guidelines (ACTION-GWTG) database in United States [16], most of the other studies from different countries found no difference in mortality rate in acute myocardial infarction patients admitted during off-hours as compared with ones admitted during regular hours [3, 17–20]. Some studies claimed that admissions on weekends were associated with higher mortality and poor outcome compared with weekday admission for patients with acute stroke [21–25]. On the contrary, several studies found that off-hour admission was not associated with an unfavorable outcome in acute ischemic stroke patients [26–32]. As for pulmonary embolism, hospitalization on weekends seemed to be associated with a significantly higher mortality rate than hospitalization on weekdays in two publications [3, 33].

With the advancement of science and modern medical technology, treatment strategies concerning specific diseases have been reshaped substantially and clinical outcomes of patients have much improved in the past 3 decades. The introduction of endovascular aneurysm repair using stent grafts has made a major paradigm shift in the field of aortic aneurysm surgery [34]. Beyond fibrinolytic therapy, primary percutaneous coronary intervention with implantation of either drug-eluting or bare-metal stents has been the cornerstone for management of acute myocardial infarction [35]. Similarly, intravenous thrombolysis and mechanical thrombectomy have made an evolutional change for the management of acute ischemic stroke [36, 37]. Novel anticoagulant therapies, systemic thrombolytic therapy, percutaneous catheter-directed treatment, and surgical pulmonary embolectomy construct the integrated reperfusion strategies for acute pulmonary embolism [38]. Allocation or re-organization of dedicated and specialized teams that are available all the time for various cardiovascular emergencies has been a major commitment of health care organizations in these decades [39]. Day of admission is only a surrogate which reflects all the efforts that health care organizations have made to eliminate difference of health care quality between on-hours and off-hours.

Taiwan has commenced a universal NHI program, financed jointly by payroll taxes, subsidies, and individual premiums, since 1995 [40]. The Taiwan’s NHI provides a comprehensive national benefit package, which includes inpatient, outpatient, and dental care [41]. As of 2013, over 99.9% of Taiwan’s 23.4 million residents were insured [41]. Taiwan’s NHI offers all citizens timely and affordable access to needed health care on equal terms [41] and the availability of the critical care service in Taiwan has increased over time under the NHI program [42]. A significant reduction in deaths [43] and an increase in life expectancy have been achieved after the introduction of Taiwan’s NHI system [40]. Throughout this study, our findings have indicated that the weekend effect on major cardiovascular emergencies has been overcome in Taiwan’s NHI system similar to what had been achieved by different health care systems in other countries [29–31].

### Study limitations

Several limitations of our study have to be acknowledged. Firstly, as information about admission hours cannot be obtained from the Taiwan NHI claims database, we could only define the weekend admission as admission on Saturday, Sunday and national festival days without further clarification regarding working hours. Secondly, we explored four specific cardiovascular emergencies, i.e. ruptured aortic aneurysm, acute myocardial infarction, ischemic stroke, and pulmonary embolism in this study. We recommended caution in extrapolating these findings to other conditions especially non-cardiovascular emergencies in Taiwan. Lastly, we defined in-hospital mortality rather than 30-day mortality as our short-term outcome in order to reflect the high case fatality rate of the serious acute illnesses we studied.

## Conclusions

Through this large retrospective cohort analysis using the Taiwan NHI claims database, we found no difference in either short-term or long-term mortality between patients admitted on weekends and patients admitted on weekdays in four major cardiovascular emergencies, i.e. ruptured aortic aneurysm, acute myocardial infarction, ischemic stroke, and pulmonary embolism. Under Taiwan’s universal NHI system, the health care providers in Taiwan have offered all citizens timely and affordable access to needed health care without significant difference between weekends and weekdays.

## Supplementary Information

**Additional file 1 Table S1.** Background characteristics of patients enrolled in ruptured aortic aneurysm subset.

**Additional file 2 Table S2.** Background characteristics of patients enrolled in acute myocardial infarction subset.

**Additional file 3 Table S3.** Background characteristics of patients enrolled in ischemic stroke subset.

**Additional file 4 Table S4.** Background characteristics of patients enrolled in pulmonary embolism subset.

**Additional file 5 Table S5.** Relative risks concerning in-hospital mortality and one-year mortality between patients admitted on different weekdays in ruptured aortic aneurysm subset.

**Additional file 6 Table S6.** Relative risks concerning in-hospital mortality and one-year mortality between patients admitted on different weekdays in acute myocardial infarction subset.

**Additional file 7 Table S7.** Relative risks concerning in-hospital mortality and one-year mortality between patients admitted on different weekdays in ischemic stroke subset.

**Additional file 8 Table S8.** Relative risks concerning in-hospital mortality and one-year mortality between patients admitted on different weekdays in pulmonary embolism subset.

## Data Availability

The authors do not own the data underlying this study. The data that support the findings of this study are available from the Health and Welfare Data Science Center (HWDC), Ministry of Health and Welfare, Executive Yuan, Taiwan. The contact information is as follows: Address: No. 488, Sec. 6, Zhongxiao E. Rd., Nangang Dist. Taipei City 11558, Taiwan; Tel:+ 886–2–8590-6805; e-mail: stsung@mohw.gov.tw. The HWDC must review and approve all applications for use of the database. The process of data analysis has to be conducted in specific offices provided by the HWDC. Only the results of analysis are released after review by the HWDC. The database owned by the HWDC is subject to related regulations of the HWDC including payment, and so is not publicly available.

## Abbreviations

ACTION-GWTG: Acute Coronary Treatment and Intervention Outcomes Network-Get With The Guidelines
CI: Confidence interval
ICD-9-CM: International Classification of Diseases Ninth Revision Clinical Modification
NHI: National Health Insurance
OR: Odds ratio
