# Supplementary material for: Mortality of major cardiovascular emergencies among patients admitted to hospitals on weekends as compared with weekdays in Taiwan
Source: BMC Health Serv Res. 2021 May 29;21:528. doi: 10.1186/s12913-021-06553-7 (PMC8164812; doi:10.1186/s12913-021-06553-7)
Supplement: Supplementary file 1 — Additional file 1 Table S1. Background characteristics of patients enrolled in ruptured aortic aneurysm subset. [file 12913_2021_6553_MOESM1_ESM.docx]

Supplementary Table 1: Background characteristics of patients enrolled in ruptured aortic aneurysm subset

|  | Weekday Group | | Weekend Group | | |  | |
| --- | --- | --- | --- | --- | --- | --- | --- |
|  | n=2,739 | | n=1,072 | | |  | |
|  | n | % | n | % | Standardized difference | |  |
| **Characteristics of hospitals** |  |  |  |  |  | |  |
| Hospital level |  |  |  |  |  | |  |
| Tertiary center | 1358 | 49.6% | 547 | 51.0% | -0.0289 | |  |
| Regional hospital | 655 | 23.9% | 287 | 26.8% | -0.0658 | |  |
| District hospital | 726 | 26.5% | 238 | 22.2% | 0.1004 | |  |
|  |  |  |  |  |  | |  |
| Teaching hospital | 2566 | 93.7% | 1023 | 95.4% | -0.0770 | |  |
| Non-teaching hospital | 173 | 6.3% | 49 | 4.6% | 0.0770 | |  |
|  |  |  |  |  |  | |  |
| Public hospital | 870 | 31.8% | 324 | 30.2% | 0.0333 | |  |
| Private hospital | 1869 | 68.2% | 748 | 69.8% | -0.0333 | |  |
|  |  |  |  |  |  | |  |
| No. of acute beds |  |  |  |  |  | |  |
| 0~199 | 200 | 7.3% | 67 | 6.3% | 0.0419 | |  |
| 200~399 | 361 | 13.2% | 160 | 14.9% | -0.0502 | |  |
| 400~599 | 468 | 17.1% | 201 | 18.8% | -0.0434 | |  |
| ≥ 600 | 1710 | 62.4% | 644 | 60.1% | 0.0484 | |  |
|  |  |  |  |  |  | |  |
| No. of cardiovascular surgeons |  |  |  |  |  | |  |
| Mean (SD) | 4.9 (5.3) | | 4.5 (4.9) | | 0.0784 | |  |
|  |  |  |  |  |  | |  |
| Volume of open heart surgeries one year prior to index date |  |  |  |  |  | |  |
| Mean (SD) | 481.2 (459.6) | | 441.9 (408.9) | | | 0.0903 | |
|  |  |  |  |  |  | |  |
| Age of attending physician |  |  |  |  |  | |  |
| Mean (SD) | 41.5 (7.3) | | 41.4 (7.4) | | 0.0136 | |  |
|  |  |  |  |  |  | |  |
| Sex of attending physician |  |  |  |  |  | |  |
| Male | 2502 | 91.3% | 959 | 89.5% | 0.0641 | |  |
| Female | 142 | 5.2% | 74 | 6.9% | -0.0722 | |  |
| Unknown | 90 | 3.3% | 37 | 3.5% | -0.0092 | |  |
|  |  |  |  |  |  | |  |
| **Characteristics of patients** |  |  |  |  |  | |  |
| Age |  |  |  |  |  | |  |
| Mean (SD) | 73.3 (13.3) | | 73.3 (13.5) | | | 0.0000 | |
|  |  |  |  |  |  | |  |
| Sex |  |  |  |  |  | |  |
| Male | 2043 | 74.6% | 840 | 78.4% | -0.0889 | |  |
| Female | 696 | 25.4% | 232 | 21.6% | 0.0889 | |  |
|  |  |  |  |  |  | |  |
| Premium |  |  |  |  |  | |  |
| Mean (SD) | 21348.3 (22857.8) | | 20820.0 (19390.9) | | | 0.0249 | |
|  |  |  |  |  |  | |  |
| Year |  |  |  |  |  | |  |
| 2006 | 248 | 9.1% | 106 | 9.9% | -0.0285 | |  |
| 2007 | 276 | 10.1% | 97 | 9.0% | 0.0350 | |  |
| 2008 | 308 | 11.2% | 124 | 11.6% | -0.0101 | |  |
| 2009 | 319 | 11.6% | 86 | 8.0% | 0.1219 | |  |
| 2010 | 288 | 10.5% | 122 | 11.4% | -0.0277 | |  |
| 2011 | 313 | 11.4% | 140 | 13.1% | -0.0498 | |  |
| 2012 | 321 | 11.7% | 127 | 11.8% | -0.0040 | |  |
| 2013 | 299 | 10.9% | 138 | 12.9% | -0.0605 | |  |
| 2014 | 367 | 13.4% | 132 | 12.3% | 0.0324 | |  |
|  |  |  |  |  |  | |  |
| Comorbidities |  |  |  |  |  | |  |
| Congestive heart failure | 339 | 12.4% | 113 | 10.5% | 0.0577 | |  |
| Cardiac arrhythmias | 249 | 9.1% | 98 | 9.1% | -0.0018 | |  |
| Valvular disease | 167 | 6.1% | 43 | 4.0% | 0.0953 | |  |
| Peripheral vascular disorders | 506 | 18.5% | 175 | 16.3% | 0.0567 | |  |
| Hypertension, uncomplicated | 1287 | 47.0% | 515 | 48.0% | -0.0211 | |  |
| Hypertension, complicated | 610 | 22.3% | 250 | 23.3% | -0.0250 | |  |
| Paralysis | 29 | 1.1% | 11 | 1.0% | 0.0032 | |  |
| Other neurological disorders | 113 | 4.1% | 40 | 3.7% | 0.0203 | |  |
| Chronic pulmonary disease | 547 | 20.0% | 213 | 19.9% | 0.0025 | |  |
| Diabetes, uncomplicated | 412 | 15.0% | 147 | 13.7% | 0.0379 | |  |
| Diabetes, complicated | 161 | 5.9% | 54 | 5.0% | 0.0370 | |  |
| Renal failure | 316 | 11.5% | 120 | 11.2% | 0.0108 | |  |
| Liver disease | 141 | 5.1% | 57 | 5.3% | -0.0076 | |  |
| Peptic ulcer disease excluding bleeding | 319 | 11.6% | 129 | 12.0% | -0.0120 | |  |
| Solid tumor without metastasis | 187 | 6.8% | 62 | 5.8% | 0.0430 | |  |
| Rheumatoid arthritis / collagen vascular diseases | 77 | 2.8% | 38 | 3.5% | -0.0418 | |  |
| Fluid and electrolyte disorders | 76 | 2.8% | 25 | 2.3% | 0.0281 | |  |
| Depression | 90 | 3.3% | 35 | 3.3% | 0.0012 | |  |
|  |  |  |  |  |  | |  |
| No. of out-patient clinic visits one year prior to index date |  |  |  |  |  | |  |
| Mean (SD) | 30.6 (23.5) | | 30.1 (23.4) | | | 0.0213 | |
|  |  |  |  |  |  | |  |
| No. of hospitalizations one year prior to index date |  |  |  |  |  | |  |
| Mean (SD) | 0.9 (1.6) | | 0.7 (1.3) | | 0.1372 | |  |
|  |  |  |  |  |  | |  |
| Medications used one year prior to index date |  |  |  |  |  | |  |
| Antiplatelet | 913 | 33.3% | 334 | 31.2% | 0.0466 | |  |
| Anticoagulant | 362 | 13.2% | 120 | 11.2% | 0.0618 | |  |
| Epilepsy | 197 | 7.2% | 61 | 5.7% | 0.0612 | |  |
| Hypertension | 832 | 30.4% | 293 | 27.3% | 0.0672 | |  |
| Tuberculosis | 56 | 2.0% | 11 | 1.0% | 0.0829 | |  |
| Rheumatic conditions | 1111 | 40.6% | 420 | 39.2% | 0.0283 | |  |
| Hyperlipidemia | 673 | 24.6% | 287 | 26.8% | -0.0504 | |  |
| Malignancies | 57 | 2.1% | 22 | 2.1% | 0.0020 | |  |
| Parkinson’s disease | 110 | 4.0% | 32 | 3.0% | 0.0561 | |  |
| Renal disease | 175 | 6.4% | 51 | 4.8% | 0.0712 | |  |
| End stage renal disease | 98 | 3.6% | 24 | 2.2% | 0.0798 | |  |
| Anti-arrhythmic | 390 | 14.2% | 124 | 11.6% | 0.0798 | |  |
| Ischemic heart disease / Angina | 860 | 31.4% | 310 | 28.9% | 0.0541 | |  |
| Congestive heart failure / Hypertension | 1613 | 58.9% | 629 | 58.7% | 0.0044 | |  |
| Diabetes | 564 | 20.6% | 203 | 18.9% | 0.0416 | |  |
| Glaucoma | 97 | 3.5% | 42 | 3.9% | -0.0199 | |  |
| Liver failure | 219 | 8.0% | 90 | 8.4% | -0.0146 | |  |
| Acid peptic disease | 1237 | 45.2% | 440 | 41.0% | 0.0832 | |  |
| Respiratory illness / asthma | 1382 | 50.5% | 505 | 47.1% | 0.0670 | |  |
| Thyroid disorders | 50 | 1.8% | 19 | 1.8% | 0.0040 | |  |
| Gout | 637 | 23.3% | 273 | 25.5% | -0.0515 | |  |
| Pain and inflammation | 2043 | 74.6% | 785 | 73.2% | 0.0310 | |  |
| Pain | 657 | 24.0% | 235 | 21.9% | 0.0491 | |  |
| Depression | 391 | 14.3% | 120 | 11.2% | 0.0925 | |  |
| Psychotic illness | 460 | 16.8% | 161 | 15.0% | 0.0486 | |  |
| Anxiety and tension | 1167 | 42.6% | 422 | 39.4% | 0.0659 | |  |
| Ischemic heart disease / Hypertension | 1905 | 69.6% | 747 | 69.7% | -0.0029 | |  |
|  |  |  |  |  |  | |  |
| Hospital transfer |  |  |  |  |  | |  |
| No | 2300 | 84.0% | 885 | 82.6% | 0.0379 | |  |
| Yes | 439 | 16.0% | 187 | 17.4% | -0.0379 | |  |
|  |  |  |  |  |  | |  |
| Surgical repair |  |  |  |  |  | |  |
| No | 1547 | 56.5% | 581 | 54.2% | 0.0459 | |  |
| Yes | 1192 | 43.5% | 491 | 45.8% | -0.0459 | |  |
|  |  |  |  |  |  | |  |
| In-hospital mortality | 1,242 | 45.3% | 487 | 45.4% | -0.0017 | |  |
| One-year mortality | 1,705 | 62.2% | 672 | 62.7% | -0.0090 | |  |

Abbreviation: SD, standard deviation
