# Supplementary material for: Mortality of major cardiovascular emergencies among patients admitted to hospitals on weekends as compared with weekdays in Taiwan
Source: BMC Health Serv Res. 2021 May 29;21:528. doi: 10.1186/s12913-021-06553-7 (PMC8164812; doi:10.1186/s12913-021-06553-7)
Supplement: Supplementary file 2 — Additional file 2 Table S2. Background characteristics of patients enrolled in acute myocardial infarction subset. [file 12913_2021_6553_MOESM2_ESM.docx]

Supplementary Table 2: Background characteristics of patients enrolled in acute myocardial infarction subset

|  | Weekday Group | | Weekend Group | |  |
| --- | --- | --- | --- | --- | --- |
|  | n=130,908 | | n=53,861 | |  |
|  | n | % | n | % | Standardized difference |
| **Characteristics of hospitals** |  |  |  |  |  |
| Hospital level |  |  |  |  |  |
| Tertiary center | 54486 | 41.6% | 22439 | 41.7% | -0.0008 |
| Regional hospital | 36855 | 28.2% | 15064 | 28.0% | 0.0041 |
| District hospital | 39567 | 30.2% | 16358 | 30.4% | -0.0032 |
|  |  |  |  |  |  |
| Teaching hospital | 117824 | 90.0% | 48502 | 90.1% | -0.0015 |
| Non-teaching hospital | 13084 | 10.0% | 5359 | 9.9% | 0.0015 |
|  |  |  |  |  |  |
| Public hospital | 37474 | 28.6% | 14722 | 27.3% | 0.0288 |
| Private hospital | 93434 | 71.4% | 39139 | 72.7% | -0.0288 |
|  |  |  |  |  |  |
| No. of acute beds |  |  |  |  |  |
| 0~199 | 15042 | 11.5% | 6343 | 11.8% | -0.0089 |
| 200~399 | 27298 | 20.9% | 11178 | 20.8% | 0.0024 |
| 400~599 | 24326 | 18.6% | 10102 | 18.8% | -0.0044 |
| ≥ 600 | 64242 | 49.1% | 26238 | 48.7% | 0.0072 |
|  |  |  |  |  |  |
| No. of cardiologists |  |  |  |  |  |
| Mean (SD) | 10.4 (9.1) | | 10.2 (9.0) | | 0.0221 |
|  |  |  |  |  |  |
| Volume of cardiac catheterizations one year prior to index date |  |  |  |  |  |
| Mean (SD) | 1220.0 (1559.6) | | 1150.5 (1492.0) | | 0.0455 |
|  | |  |  |  |  |
| Age of attending physician |  |  |  |  |  |
| Mean (SD) | 41.9 (7.9) | | 41.6 (7.8) | | 0.0382 |
|  |  |  |  |  |  |
| Sex of attending physician |  |  |  |  |  |
| Male | 119570 | 91.3% | 48927 | 90.8% | 0.0175 |
| Female | 7531 | 5.8% | 3188 | 5.9% | -0.0071 |
| Unknown | 3807 | 2.9% | 1746 | 3.2% | -0.0193 |
|  |  |  |  |  |  |
| **Characteristics of patients** |  |  |  |  |  |
| Age |  |  |  |  |  |
| Mean (SD) | 68.1 (14.5) | | 67.9 (14.6) | | 0.0137 |
|  |  |  |  |  |  |
| Sex |  |  |  |  |  |
| Male | 89658 | 68.5% | 36748 | 68.2% | 0.0056 |
| Female | 41250 | 31.5% | 17113 | 31.8% | -0.0056 |
|  |  |  |  |  |  |
| Premium |  |  |  |  |  |
| Mean (SD) | 21664.8 (21000.3) | | 22080.8 (21210.8) | | -0.0197 |
|  |  |  |  |  |  |
| Year |  |  |  |  |  |
| 2006 | 13100 | 10.0% | 5366 | 10.0% | 0.0015 |
| 2007 | 13588 | 10.4% | 5656 | 10.5% | -0.0040 |
| 2008 | 14096 | 10.8% | 5632 | 10.5% | 0.0101 |
| 2009 | 13700 | 10.5% | 5688 | 10.6% | -0.0031 |
| 2010 | 14391 | 11.0% | 5834 | 10.8% | 0.0052 |
| 2011 | 14615 | 11.2% | 6189 | 11.5% | -0.0103 |
| 2012 | 15544 | 11.9% | 6231 | 11.6% | 0.0095 |
| 2013 | 15780 | 12.1% | 6555 | 12.2% | -0.0036 |
| 2014 | 16094 | 12.3% | 6710 | 12.5% | -0.0050 |
|  |  |  |  |  |  |
| Comorbidities |  |  |  |  |  |
| Congestive heart failure | 16537 | 12.6% | 6435 | 11.9% | 0.0209 |
| Cardiac arrhythmias | 11295 | 8.6% | 4470 | 8.3% | 0.0118 |
| Valvular disease | 5217 | 4.0% | 2144 | 4.0% | 0.0002 |
| Peripheral vascular disorders | 3900 | 3.0% | 1510 | 2.8% | 0.0105 |
| Hypertension, uncomplicated | 58880 | 45.0% | 23779 | 44.1% | 0.0167 |
| Hypertension, complicated | 25224 | 19.3% | 10131 | 18.8% | 0.0117 |
| Other neurological disorders | 4850 | 3.7% | 1846 | 3.4% | 0.0150 |
| Chronic pulmonary disease | 20039 | 15.3% | 7952 | 14.8% | 0.0152 |
| Diabetes, uncomplicated | 37769 | 28.9% | 15271 | 28.4% | 0.0110 |
| Diabetes, complicated | 19614 | 15.0% | 7795 | 14.5% | 0.0144 |
| Renal failure | 15632 | 11.9% | 5942 | 11.0% | 0.0285 |
| Liver disease | 8006 | 6.1% | 3143 | 5.8% | 0.0118 |
| Peptic ulcer disease excluding bleeding | 12809 | 9.8% | 5039 | 9.4% | 0.0146 |
| Solid tumor without metastasis | 7333 | 5.6% | 2774 | 5.2% | 0.0200 |
| Rheumatoid arthritis / collagen vascular diseases | 3212 | 2.5% | 1304 | 2.4% | 0.0021 |
| Fluid and electrolyte disorders | 3506 | 2.7% | 1312 | 2.4% | 0.0154 |
| Blood loss anemia or deficiency anemia | 1631 | 1.2% | 657 | 1.2% | 0.0024 |
| Depression | 4247 | 3.2% | 1744 | 3.2% | 0.0004 |
|  |  |  |  |  |  |
| No. of out-patient clinic visits one year prior to index date |  |  |  |  |  |
| Mean (SD) | 29.5 | 24.8 | 28.8 | 24.8 | 0.0282 |
|  |  |  |  |  |  |
| No. of hospitalizations one year prior to index date |  |  |  |  |  |
| Mean (SD) | 0.7 | 1.4 | 0.6 | 1.3 | 0.0740 |
|  |  |  |  |  |  |
| Medications used one year prior to index date |  |  |  |  |  |
| Antiplatelet | 42337 | 32.3% | 16541 | 30.7% | 0.0351 |
| Anticoagulant | 16089 | 12.3% | 5803 | 10.8% | 0.0475 |
| Epilepsy | 7905 | 6.0% | 3150 | 5.8% | 0.0080 |
| Hypertension | 32290 | 24.7% | 12853 | 23.9% | 0.0187 |
| Tuberculosis | 1339 | 1.0% | 511 | 0.9% | 0.0075 |
| Rheumatic conditions | 43388 | 33.1% | 17460 | 32.4% | 0.0155 |
| Hyperlipidemia | 37565 | 28.7% | 14996 | 27.8% | 0.0190 |
| Malignancies | 2574 | 2.0% | 974 | 1.8% | 0.0116 |
| Parkinson’s disease | 4998 | 3.8% | 1943 | 3.6% | 0.0111 |
| Renal disease | 8302 | 6.3% | 3043 | 5.6% | 0.0292 |
| End stage renal disease | 7648 | 5.8% | 2694 | 5.0% | 0.0371 |
| Anti-arrhythmic | 16293 | 12.4% | 6320 | 11.7% | 0.0218 |
| Ischemic heart disease / Angina | 45485 | 34.7% | 17240 | 32.0% | 0.0581 |
| Congestive heart failure / Hypertension | 72529 | 55.4% | 28744 | 53.4% | 0.0409 |
| Diabetes | 46992 | 35.9% | 18867 | 35.0% | 0.0181 |
| Glaucoma | 5369 | 4.1% | 2046 | 3.8% | 0.0155 |
| Liver failure | 6243 | 4.8% | 2397 | 4.5% | 0.0152 |
| Acid peptic disease | 48949 | 37.4% | 19515 | 36.2% | 0.0240 |
| Respiratory illness / asthma | 58036 | 44.3% | 23555 | 43.7% | 0.0121 |
| Thyroid disorders | 2154 | 1.6% | 842 | 1.6% | 0.0065 |
| Gout | 22904 | 17.5% | 9141 | 17.0% | 0.0139 |
| Pain and inflammation | 90648 | 69.2% | 37104 | 68.9% | 0.0077 |
| Pain | 20517 | 15.7% | 7854 | 14.6% | 0.0304 |
| Depression | 15478 | 11.8% | 6257 | 11.6% | 0.0064 |
| Psychotic illness | 19627 | 15.0% | 7711 | 14.3% | 0.0191 |
| Anxiety and tension | 49383 | 37.7% | 19767 | 36.7% | 0.0212 |
| Ischemic heart disease / Hypertension | 82493 | 63.0% | 32849 | 61.0% | 0.0418 |
|  |  |  |  |  |  |
| Type of myocardial infarction |  |  |  |  |  |
| STEMI | 30388 | 23.2% | 12566 | 23.3% | -0.0028 |
| NSTEMI | 100520 | 76.8% | 41295 | 76.7% | 0.0028 |
|  |  |  |  |  |  |
| Hospital transfer |  |  |  |  |  |
| No | 90821 | 69.4% | 35870 | 66.6% | 0.0596 |
| Yes | 40087 | 30.6% | 17991 | 33.4% | -0.0596 |
|  |  |  |  |  |  |
| Percutaneous coronary intervention |  |  |  |  |  |
| No | 69997 | 53.5% | 29049 | 53.9% | -0.0093 |
| Yes | 60911 | 46.5% | 24812 | 46.1% | 0.0093 |
|  |  |  |  |  |  |
| Fibrinolytic therapy |  |  |  |  |  |
| No | 128559 | 98.2% | 52745 | 97.9% | 0.0202 |
| Yes | 2349 | 1.8% | 1116 | 2.1% | -0.0202 |
|  |  |  |  |  |  |
| In-hospital mortality | 21211 | 16.2% | 8494 | 15.8% | 0.0118 |
| One-year mortality | 40480 | 30.9% | 16248 | 30.2% | 0.0164 |

Abbreviations: NSTEMI, non-ST elevation myocardial infarction; SD, standard deviation; STEMI, ST elevation myocardial infarction
