# Supplementary material for: Mortality of major cardiovascular emergencies among patients admitted to hospitals on weekends as compared with weekdays in Taiwan
Source: BMC Health Serv Res. 2021 May 29;21:528. doi: 10.1186/s12913-021-06553-7 (PMC8164812; doi:10.1186/s12913-021-06553-7)
Supplement: Supplementary file 3 — Additional file 3 Table S3. Background characteristics of patients enrolled in ischemic stroke subset. [file 12913_2021_6553_MOESM3_ESM.docx]

Supplementary Table 3: Background characteristics of patients enrolled in ischemic stroke subset

|  | Weekday Group | | Weekend Group | |  |
| --- | --- | --- | --- | --- | --- |
|  | n=355,400 | | n=136,727 | |  |
|  | n | % | n | % | Standardized difference |
| **Characteristics of hospitals** |  |  |  |  |  |
| Hospital level |  |  |  |  |  |
| Tertiary center | 130,170 | 36.6% | 50,908 | 37.2% | -0.0126 |
| Regional hospital | 101,952 | 28.7% | 40,314 | 29.5% | -0.0176 |
| District hospital | 123,278 | 34.7% | 45,505 | 33.3% | 0.0297 |
|  |  |  |  |  |  |
| Teaching hospital | 308,465 | 86.8% | 120,617 | 88.2% | -0.0431 |
| Non-teaching hospital | 46,935 | 13.2% | 16,110 | 11.8% | 0.0431 |
|  |  |  |  |  |  |
| Public hospital | 98,709 | 27.8% | 36,333 | 26.6% | 0.0270 |
| Private hospital | 256,691 | 72.2% | 100,394 | 73.4% | -0.0270 |
|  |  |  |  |  |  |
| No. of acute beds |  |  |  |  |  |
| 0~199 | 52,229 | 14.7% | 18,231 | 13.3% | 0.0392 |
| 200~399 | 79,426 | 22.3% | 30,830 | 22.5% | -0.0048 |
| 400~599 | 65,358 | 18.4% | 25,900 | 18.9% | -0.0142 |
| ≥ 600 | 158,387 | 44.6% | 61,766 | 45.2% | -0.0122 |
|  |  |  |  |  |  |
| No. of neurologists |  |  |  |  |  |
| mean (SD) | 7.5 (8.0) | | 7.6 (8.0) | | -0.0038 |
|  |  |  |  |  |  |
| Age of attending physician |  |  |  |  |  |
| mean (SD) | 42.3 (8.3) | | 42.0 (8.2) | | 0.0352 |
|  |  |  |  |  |  |
| Sex of attending physician |  |  |  |  |  |
| Male | 306,000 | 86.1% | 117,257 | 85.8% | 0.0098 |
| Female | 43,129 | 12.1% | 16,721 | 12.2% | -0.0029 |
| Unknown | 6,271 | 1.8% | 2,794 | 2.0% | -0.0204 |
|  |  |  |  |  |  |
| **Characteristics of patients** |  |  |  |  |  |
| Age |  |  |  |  |  |
| mean (SD) | 68.6 (14.1) | | 68.5 (14.0) | | 0.0071 |
|  |  |  |  |  |  |
| Sex |  |  |  |  |  |
| Male | 205,097 | 57.7% | 79,041 | 57.8% | -0.0020 |
| Female | 150,303 | 42.3% | 57,686 | 42.2% | 0.0020 |
|  |  |  |  |  |  |
| Premium |  |  |  |  |  |
| mean (SD) | 20890.5 (19553.3) | | 21093.8 (19356.5) | | -0.0104 |
|  |  |  |  |  |  |
| Year |  |  |  |  |  |
| 2006 | 43,196 | 12.2% | 16,290 | 11.9% | 0.0074 |
| 2007 | 42,067 | 11.8% | 15,851 | 11.6% | 0.0076 |
| 2008 | 40,170 | 11.3% | 15,398 | 11.3% | 0.0013 |
| 2009 | 39,596 | 11.1% | 14,802 | 10.8% | 0.0101 |
| 2010 | 39,525 | 11.1% | 15,099 | 11.0% | 0.0025 |
| 2011 | 38,789 | 10.9% | 15,354 | 11.2% | -0.0101 |
| 2012 | 38,297 | 10.8% | 14,680 | 10.7% | 0.0013 |
| 2013 | 37,132 | 10.4% | 14,803 | 10.8% | -0.0123 |
| 2014 | 36,628 | 10.3% | 14,450 | 10.6% | -0.0086 |
|  |  |  |  |  |  |
| Comorbidities |  |  |  |  |  |
| Congestive heart failure | 28,920 | 8.1% | 11,021 | 8.1% | 0.0028 |
| Cardiac arrhythmias | 29,920 | 8.4% | 11,663 | 8.5% | -0.0040 |
| Valvular disease | 11,944 | 3.4% | 4,592 | 3.4% | 0.0001 |
| Peripheral vascular disorders | 8,667 | 2.4% | 3,197 | 2.3% | 0.0066 |
| Hypertension, uncomplicated | 154,849 | 43.6% | 59,041 | 43.2% | 0.0078 |
| Hypertension, complicated | 58,727 | 16.5% | 21,986 | 16.1% | 0.0120 |
| Other neurological disorders | 16,357 | 4.6% | 5,899 | 4.3% | 0.0140 |
| Chronic pulmonary disease | 44,547 | 12.5% | 16,574 | 12.1% | 0.0125 |
| Diabetes, uncomplicated | 87,855 | 24.7% | 32,915 | 24.1% | 0.0151 |
| Diabetes, complicated | 40,534 | 11.4% | 14,861 | 10.9% | 0.0170 |
| Renal failure | 21,395 | 6.0% | 7,632 | 5.6% | 0.0187 |
| Liver disease | 23,532 | 6.6% | 8,849 | 6.5% | 0.0060 |
| Peptic ulcer disease excluding bleeding | 32,401 | 9.1% | 11,950 | 8.7% | 0.0132 |
| Solid tumor without metastasis | 20,355 | 5.7% | 7,543 | 5.5% | 0.0091 |
| Rheumatoid arthritis / collagen vascular diseases | 8,250 | 2.3% | 3,049 | 2.2% | 0.0061 |
| Fluid and electrolyte disorders | 7,025 | 2.0% | 2,455 | 1.8% | 0.0133 |
| Blood loss anemia or deficiency anemia | 3,453 | 1.0% | 1,341 | 1.0% | -0.0009 |
| Psychoses | 4,169 | 1.2% | 1,560 | 1.1% | 0.0030 |
| Depression | 15,032 | 4.2% | 5,537 | 4.0% | 0.0090 |
|  |  |  |  |  |  |
| No. of out-patient clinic visits one year prior to index date |  |  |  |  |  |
| mean (SD) | 27.7 (24.0) | | 27.1 (23.8) | | 0.0238 |
|  |  |  |  |  |  |
| No. of hospitalizations one year prior to index date |  |  |  |  |  |
| mean (SD) | 0.5 (1.2) | | 0.5 (1.1) | | 0.0423 |
|  |  |  |  |  |  |
| Medications used one year prior to index date |  |  |  |  |  |
| Antiplatelet | 81,208 | 22.8% | 29,639 | 21.7% | 0.0282 |
| Anticoagulant | 22,751 | 6.4% | 8,174 | 6.0% | 0.0176 |
| Epilepsy | 24,819 | 7.0% | 8,837 | 6.5% | 0.0208 |
| Hypertension | 77,784 | 21.9% | 28,927 | 21.2% | 0.0178 |
| Rheumatic conditions | 106,535 | 30.0% | 39,860 | 29.2% | 0.0180 |
| Hyperlipidemia | 75,149 | 21.1% | 28,366 | 20.7% | 0.0098 |
| Malignancies | 6,963 | 2.0% | 2,506 | 1.8% | 0.0093 |
| Parkinson’s disease | 15,599 | 4.4% | 5,691 | 4.2% | 0.0112 |
| Renal disease | 10,641 | 3.0% | 3,655 | 2.7% | 0.0193 |
| End stage renal disease | 8,610 | 2.4% | 2,821 | 2.1% | 0.0243 |
| Anti-arrhythmic | 34,402 | 9.7% | 12,937 | 9.5% | 0.0074 |
| Ischemic heart disease / Angina | 62,565 | 17.6% | 22,978 | 16.8% | 0.0212 |
| Congestive heart failure / Hypertension | 172,848 | 48.6% | 65,307 | 47.8% | 0.0174 |
| Diabetes | 109,299 | 30.8% | 40,710 | 29.8% | 0.0213 |
| Glaucoma | 14,683 | 4.1% | 5,295 | 3.9% | 0.0132 |
| Liver failure | 12,607 | 3.5% | 4,265 | 3.1% | 0.0238 |
| Acid peptic disease | 119,988 | 33.8% | 45,085 | 33.0% | 0.0167 |
| Respiratory illness / asthma | 138,295 | 38.9% | 52,714 | 38.6% | 0.0074 |
| Thyroid disorders | 6,059 | 1.7% | 2,372 | 1.7% | -0.0023 |
| Gout | 48,651 | 13.7% | 18,756 | 13.7% | -0.0008 |
| Pain and inflammation | 247,847 | 69.7% | 94,635 | 69.2% | 0.0114 |
| Pain | 38,950 | 11.0% | 14,126 | 10.3% | 0.0204 |
| Depression | 46,545 | 13.1% | 16,959 | 12.4% | 0.0208 |
| Psychotic illness | 58,834 | 16.6% | 20,953 | 15.3% | 0.0336 |
| Anxiety and tension | 139,831 | 39.3% | 51,457 | 37.6% | 0.0351 |
| Ischemic heart disease / Hypertension | 213,867 | 60.2% | 80,832 | 59.1% | 0.0215 |
|  |  |  |  |  |  |
| Hospital transfer |  |  |  |  |  |
| No | 270,031 | 76.0% | 99,890 | 73.1% | 0.0671 |
| Yes | 85,369 | 24.0% | 36,837 | 26.9% | -0.0671 |
|  |  |  |  |  |  |
| Fibrinolytic therapy |  |  |  |  |  |
| No | 349,592 | 98.4% | 134,281 | 98.2% | 0.0119 |
| Yes | 5,808 | 1.6% | 2,446 | 1.8% | -0.0119 |
|  |  |  |  |  |  |
| In-hospital mortality | 14,778 | 4.2% | 5,567 | 4.1% | 0.0044 |
| One-year mortality | 57,458 | 16.2% | 21,378 | 15.6% | 0.0145 |

Abbreviation: SD, standard deviation
