# Supplementary material for: Mortality of major cardiovascular emergencies among patients admitted to hospitals on weekends as compared with weekdays in Taiwan
Source: BMC Health Serv Res. 2021 May 29;21:528. doi: 10.1186/s12913-021-06553-7 (PMC8164812; doi:10.1186/s12913-021-06553-7)
Supplement: Supplementary file 4 — Additional file 4 Table S4. Background characteristics of patients enrolled in pulmonary embolism subset. [file 12913_2021_6553_MOESM4_ESM.docx]

Supplementary Table 4: Background characteristics of patients enrolled in pulmonary embolism subset

|  | Weekday Group | | Weekend Group | |  |
| --- | --- | --- | --- | --- | --- |
|  | n=11,527 | | n=3,506 | |  |
|  | n | % | n | % | Standardized difference |
| **Characteristics of hospitals** |  |  |  |  |  |
| Hospital level |  |  |  |  |  |
| Tertiary center | 5744 | 49.8% | 1833 | 52.3% | -0.0490 |
| Regional hospital | 2950 | 25.6% | 848 | 24.2% | 0.0325 |
| District hospital | 2833 | 24.6% | 825 | 23.5% | 0.0245 |
|  |  |  |  |  |  |
| Teaching hospital | 10982 | 95.3% | 3394 | 96.8% | -0.0787 |
| Non-teaching hospital | 545 | 4.7% | 112 | 3.2% | 0.0787 |
|  |  |  |  |  |  |
| Public hospital | 3479 | 30.2% | 957 | 27.3% | 0.0638 |
| Private hospital | 8048 | 69.8% | 2549 | 72.7% | -0.0638 |
|  |  |  |  |  |  |
| No. of acute beds |  |  |  |  |  |
| 0~199 | 661 | 5.7% | 160 | 4.6% | 0.0530 |
| 200~399 | 1647 | 14.3% | 430 | 12.3% | 0.0597 |
| 400~599 | 2009 | 17.4% | 582 | 16.6% | 0.0221 |
| ≥ 600 | 7210 | 62.5% | 2334 | 66.6% | -0.0842 |
|  |  |  |  |  |  |
| No. of cardiologists and cardiovascular surgeons |  |  |  |  |  |
| Mean (SD) | 17.6 (15.2) | | 18.0 (14.8) | | -0.0267 |
|  |  |  |  |  |  |
| Volume of surgical or trans-catheter embolectomies one year prior to index date |  |  |  |  |  |
| Mean (SD) | 194.4 (301.0) | | 184.9 (260.1) | | 0.0338 |
|  |  |  |  |  |  |
| Age of attending physician |  |  |  |  |  |
| Mean (SD) | 43.4 (7.8) | | 42.9 (7.7) | | 0.0645 |
|  |  |  |  |  |  |
| Sex of attending physician |  |  |  |  |  |
| Male | 10685 | 92.7% | 3223 | 91.9% | 0.0288 |
| Female | 700 | 6.1% | 226 | 6.4% | -0.0154 |
| Unknown | 142 | 1.2% | 57 | 1.6% | -0.0332 |
|  |  |  |  |  |  |
| **Characteristics of patients** |  |  |  |  |  |
| Age |  |  |  |  |  |
| Mean (SD) | 67.9 | 16.1 | 68.3 | 16.3 | -0.0247 |
|  |  |  |  |  |  |
| Sex |  |  |  |  |  |
| Male | 5282 | 45.8% | 1576 | 45.0% | 0.0175 |
| Female | 6245 | 54.2% | 1930 | 55.0% | -0.0175 |
|  |  |  |  |  |  |
| Premium |  |  |  |  |  |
| Mean (SD) | 21918.0 (20735.3) | | 21897.0 (20432.3) | | 0.0010 |
|  |  |  |  |  |  |
| Year |  |  |  |  |  |
| 2006 | 948 | 8.2% | 286 | 8.2% | 0.0024 |
| 2007 | 1045 | 9.1% | 332 | 9.5% | -0.0139 |
| 2008 | 1202 | 10.4% | 356 | 10.2% | 0.0090 |
| 2009 | 1306 | 11.3% | 398 | 11.4% | -0.0007 |
| 2010 | 1319 | 11.4% | 396 | 11.3% | 0.0047 |
| 2011 | 1414 | 12.3% | 424 | 12.1% | 0.0053 |
| 2012 | 1344 | 11.7% | 404 | 11.5% | 0.0043 |
| 2013 | 1389 | 12.0% | 444 | 12.7% | -0.0187 |
| 2014 | 1560 | 13.5% | 466 | 13.3% | 0.0071 |
|  |  |  |  |  |  |
| Comorbidities |  |  |  |  |  |
| Congestive heart failure | 1,865 | 16.2% | 612 | 17.5% | -0.0341 |
| Cardiac arrhythmias | 1,461 | 12.7% | 437 | 12.5% | 0.0063 |
| Valvular disease | 703 | 6.1% | 214 | 6.1% | -0.0002 |
| Pulmonary circulation disorders | 142 | 1.2% | 42 | 1.2% | 0.0031 |
| Peripheral vascular disorders | 351 | 3.0% | 115 | 3.3% | -0.0134 |
| Hypertension, uncomplicated | 4,770 | 41.4% | 1,475 | 42.1% | -0.0140 |
| Hypertension, complicated | 2,028 | 17.6% | 610 | 17.4% | 0.0051 |
| Paralysis | 156 | 1.4% | 48 | 1.4% | -0.0014 |
| Other neurological disorders | 613 | 5.3% | 188 | 5.4% | -0.0020 |
| Chronic pulmonary disease | 2,830 | 24.6% | 796 | 22.7% | 0.0435 |
| Diabetes, uncomplicated | 2,293 | 19.9% | 713 | 20.3% | -0.0111 |
| Diabetes, complicated | 938 | 8.1% | 302 | 8.6% | -0.0172 |
| Hypothyroidism | 150 | 1.3% | 44 | 1.3% | 0.0041 |
| Renal failure | 845 | 7.3% | 223 | 6.4% | 0.0384 |
| Liver disease | 915 | 7.9% | 278 | 7.9% | 0.0003 |
| Peptic ulcer disease excluding bleeding | 1,451 | 12.6% | 452 | 12.9% | -0.0091 |
| Metastatic cancer | 876 | 7.6% | 247 | 7.0% | 0.0213 |
| Solid tumor without metastasis | 2,177 | 18.9% | 598 | 17.1% | 0.0477 |
| Rheumatoid arthritis / collagen vascular diseases | 534 | 4.6% | 148 | 4.2% | 0.0200 |
| Weight loss | 142 | 1.2% | 38 | 1.1% | 0.0138 |
| Fluid and electrolyte disorders | 410 | 3.6% | 139 | 4.0% | -0.0214 |
| Blood loss anemia or deficiency anemia | 220 | 1.9% | 76 | 2.2% | -0.0183 |
| Psychoses | 177 | 1.5% | 39 | 1.1% | 0.0370 |
| Depression | 640 | 5.6% | 201 | 5.7% | -0.0078 |
|  |  |  |  |  |  |
| No. of out-patient clinic visits one year prior to index date |  |  |  |  |  |
| Mean (SD) | 36.1 (25.8) | | 35.7 (25.3) | | 0.0157 |
|  |  |  |  |  |  |
| No. of hospitalizations one year prior to index date |  |  |  |  |  |
| Mean (SD) | 1.4 (2.3) | | 1.3 (2.1) | | 0.0454 |
|  |  |  |  |  |  |
| Medications used one year prior to index date |  |  |  |  |  |
| Antiplatelet | 3,274 | 28.4% | 965 | 27.5% | 0.0196 |
| Anticoagulant | 2,889 | 25.1% | 820 | 23.4% | 0.0391 |
| Epilepsy | 1,103 | 9.6% | 307 | 8.8% | 0.0282 |
| Hypertension | 3,429 | 29.7% | 1,030 | 29.4% | 0.0081 |
| Tuberculosis | 179 | 1.6% | 47 | 1.3% | 0.0178 |
| Rheumatic conditions | 5,996 | 52.0% | 1,766 | 50.4% | 0.0329 |
| Hyperlipidemia | 2,480 | 21.5% | 740 | 21.1% | 0.0100 |
| Malignancies | 1,453 | 12.6% | 381 | 10.9% | 0.0540 |
| Parkinson’s disease | 609 | 5.3% | 185 | 5.3% | 0.0003 |
| Renal disease | 651 | 5.6% | 201 | 5.7% | -0.0037 |
| End stage renal disease | 247 | 2.1% | 76 | 2.2% | -0.0017 |
| Anti-arrhythmic | 2,123 | 18.4% | 621 | 17.7% | 0.0183 |
| Ischemic heart disease / Angina | 3,424 | 29.7% | 1,047 | 29.9% | -0.0035 |
| Congestive heart failure / Hypertension | 7,212 | 62.6% | 2,150 | 61.3% | 0.0256 |
| Diabetes | 2,876 | 25.0% | 880 | 25.1% | -0.0035 |
| Glaucoma | 539 | 4.7% | 154 | 4.4% | 0.0136 |
| Liver failure | 912 | 7.9% | 261 | 7.4% | 0.0176 |
| Acid peptic disease | 5,679 | 49.3% | 1,672 | 47.7% | 0.0316 |
| Respiratory illness / asthma | 6,653 | 57.7% | 2,007 | 57.2% | 0.0095 |
| Thyroid disorders | 318 | 2.8% | 96 | 2.7% | 0.0013 |
| Gout | 1,913 | 16.6% | 570 | 16.3% | 0.0091 |
| Pain and inflammation | 9,141 | 79.3% | 2,817 | 80.3% | -0.0261 |
| Pain | 3,414 | 29.6% | 1,062 | 30.3% | -0.0147 |
| Depression | 2,021 | 17.5% | 616 | 17.6% | -0.0010 |
| Psychotic illness | 2,757 | 23.9% | 855 | 24.4% | -0.0110 |
| Anxiety and tension | 5,968 | 51.8% | 1,806 | 51.5% | 0.0053 |
| Ischemic heart disease / Hypertension | 7,365 | 63.9% | 2,194 | 62.6% | 0.0273 |
|  |  |  |  |  |  |
| Hospital transfer |  |  |  |  |  |
| No | 9910 | 86.0% | 2999 | 85.5% | 0.0124 |
| Yes | 1617 | 14.0% | 507 | 14.5% | -0.0124 |
|  |  |  |  |  |  |
| Anti-coagulant therapy | 11467 | 99.5% | 3486 | 99.4% | 0.0068 |
| Fibrinolytic therapy | 895 | 7.8% | 265 | 7.6% | 0.0077 |
| Surgical embolectomy | 129 | 1.1% | 30 | 0.9% | 0.0266 |
| Trans-catheter embolectomy | 72 | 0.6% | 17 | 0.5% | 0.0188 |
|  |  |  |  |  |  |
| In-hospital mortality | 1,683 | 14.6% | 513 | 14.6% | -0.0009 |
| One-year mortality | 4,075 | 35.4% | 1,240 | 35.4% | -0.0003 |

Abbreviation: SD, standard deviation
