# Supplementary material for: Mortality of major cardiovascular emergencies among patients admitted to hospitals on weekends as compared with weekdays in Taiwan
Source: BMC Health Serv Res. 2021 May 29;21:528. doi: 10.1186/s12913-021-06553-7 (PMC8164812; doi:10.1186/s12913-021-06553-7)
Supplement: Supplementary file 5 — Additional file 5 Table S5. Relative risks concerning in-hospital mortality and one-year mortality between patients admitted on different weekdays in ruptured aortic aneurysm subset. [file 12913_2021_6553_MOESM5_ESM.docx]

Supplementary Table 5: Relative risks concerning in-hospital mortality and one-year mortality between patients admitted on different weekdays in ruptured aortic aneurysm subset.

| In-hospital mortality | | | |  |  |  |  |  |
| --- | --- | --- | --- | --- | --- | --- | --- | --- |
|  |  | Reference Day | | | | | | |
|  | OR  (95% CI) | Sunday | Monday | Tuesday | Wednesday | Thursday | Friday | Saturday |
|  | Sunday | 1 | 1.149  (0.879~1.500) | 1.235  (0.941~1.621) | 1.061  (0.806~1.397) | 1.112  (0.849~1.456) | 1.138  (0.870~1.487) | 1.247  (0.948~1.641) |
|  | Monday |  | 1 | 1.075  (0.831~1.391) | 0.924  (0.712~1.198) | 0.968  (0.750~1.249) | 0.991  (0.769~1.277) | 1.086  (0.837~1.409) |
|  | Tuesday |  |  | 1 | 0.859  (0.659~1.120) | 0.900  (0.695~1.167) | 0.921  (0.711~1.193) | 1.010  (0.775~1.317) |
|  | Wednesday |  |  |  | 1 | 1.048  (0.806~1.363) | 1.072  (0.826~1.393) | 1.176  (0.899~1.538) |
|  | Thursday |  |  |  |  | 1 | 1.023  (0.792~1.322) | 1.122  (0.862~1.460) |
|  | Friday |  |  |  |  |  | 1 | 1.096  (0.844~1.424) |
|  | Saturday |  |  |  |  |  |  | 1 |

| One-year mortality | | | |  |  |  |  |  |
| --- | --- | --- | --- | --- | --- | --- | --- | --- |
|  |  | Reference Day | | | | | | |
|  | OR  (95% CI) | Sunday | Monday | Tuesday | Wednesday | Thursday | Friday | Saturday |
|  | Sunday | 1 | 0.974 (0.736~1.290) | 1.088 (0.819~1.447) | 1.011 (0.758~1.349) | 1.106 (0.834~1.468) | 0.967 (0.729~1.283) | 0.962 (0.720~1.286) |
|  | Monday |  | 1 | 1.117 (0.853~1.463) | 1.038 (0.790~1.363) | 1.136 (0.869~1.484) | 0.993 (0.759~1.298) | 0.988 (0.750~1.301) |
|  | Tuesday |  |  | 1 | 0.929 (0.704~1.226) | 1.017 (0.776~1.332) | 0.889 (0.677~1.167) | 0.884 (0.669~1.169) |
|  | Wednesday |  |  |  | 1 | 1.094 (0.831~1.441) | 0.956 (0.726~1.261) | 0.952 (0.717~1.263) |
|  | Thursday |  |  |  |  | 1 | 0.874 (0.667~1.145) | 0.870 (0.658~1.149) |
|  | Friday |  |  |  |  |  | 1 | 0.995 (0.753~1.315) |
|  | Saturday |  |  |  |  |  |  | 1 |

Abbreviations: CI, confidence interval; OR, odds ratio.
