# Supplementary material for: Mortality of major cardiovascular emergencies among patients admitted to hospitals on weekends as compared with weekdays in Taiwan
Source: BMC Health Serv Res. 2021 May 29;21:528. doi: 10.1186/s12913-021-06553-7 (PMC8164812; doi:10.1186/s12913-021-06553-7)
Supplement: Supplementary file 6 — Additional file 6 Table S6. Relative risks concerning in-hospital mortality and one-year mortality between patients admitted on different weekdays in acute myocardial infarction subset. [file 12913_2021_6553_MOESM6_ESM.docx]

Supplementary Table 6: Relative risks concerning in-hospital mortality and one-year mortality between patients admitted on different weekdays in acute myocardial infarction subset.

| In-hospital mortality | | | |  |  |  |  |  |
| --- | --- | --- | --- | --- | --- | --- | --- | --- |
|  |  | Reference Day | | | | | | |
|  | OR  (95% CI) | Sunday | Monday | Tuesday | Wednesday | Thursday | Friday | Saturday |
|  | Sunday | 1 | 1.022 (0.973~1.075) | 0.994 (0.945~1.046) | 0.963 (0.915~1.013) | 0.953 (0.906~1.002) | 0.954 (0.907~1.003) | 1.000 (0.950~1.052) |
|  | Monday |  | 1 | 0.972 (0.926~1.021) | 0.941 (0.897~0.989) | 0.932 (0.888~0.978) | 0.933 (0.889~0.980) | 0.978 (0.931~1.027) |
|  | Tuesday |  |  | 1 | 0.968 (0.921~1.018) | 0.958 (0.912~1.007) | 0.960 (0.914~1.008) | 1.006 (0.957~1.058) |
|  | Wednesday |  |  |  | 1 | 0.990 (0.942~1.040) | 0.991 (0.943~1.042) | 1.039 (0.988~1.092) |
|  | Thursday |  |  |  |  | 1 | 1.002 (0.953~1.052) | 1.049 (0.998~1.103) |
|  | Friday |  |  |  |  |  | 1 | 1.048 (0.997~1.102) |
|  | Saturday |  |  |  |  |  |  | 1 |

| One-year mortality | | | |  |  |  |  |  |
| --- | --- | --- | --- | --- | --- | --- | --- | --- |
|  |  | Reference Day | | | | | | |
|  | OR  (95% CI) | Sunday | Monday | Tuesday | Wednesday | Thursday | Friday | Saturday |
|  | Sunday | 1 | 1.008 (0.966~1.052) | 0.994 (0.952~1.038) | 0.983 (0.941~1.027) | 0.973 (0.931~1.016) | 0.957 (0.916~0.999) | 0.999 (0.956~1.043) |
|  | Monday |  | 1 | 0.986 (0.946~1.028) | 0.975 (0.935~1.017) | 0.964 (0.925~1.006) | 0.949 (0.910~0.989) | 0.990 (0.949~1.033) |
|  | Tuesday |  |  | 1 | 0.989 (0.947~1.032) | 0.978 (0.937~1.021) | 0.962 (0.922~1.004) | 1.005 (0.962~1.049) |
|  | Wednesday |  |  |  | 1 | 0.989 (0.948~1.033) | 0.973 (0.932~1.016) | 1.016 (0.973~1.061) |
|  | Thursday |  |  |  |  | 1 | 0.984 (0.943~1.027) | 1.027 (0.983~1.072) |
|  | Friday |  |  |  |  |  | 1 | 1.044 (1.000~1.090) |
|  | Saturday |  |  |  |  |  |  | 1 |

Abbreviations: CI, confidence interval; OR, odds ratio.
