# Supplementary material for: Mortality of major cardiovascular emergencies among patients admitted to hospitals on weekends as compared with weekdays in Taiwan
Source: BMC Health Serv Res. 2021 May 29;21:528. doi: 10.1186/s12913-021-06553-7 (PMC8164812; doi:10.1186/s12913-021-06553-7)
Supplement: Supplementary file 7 — Additional file 7 Table S7. Relative risks concerning in-hospital mortality and one-year mortality between patients admitted on different weekdays in ischemic stroke subset. [file 12913_2021_6553_MOESM7_ESM.docx]

Supplementary Table 7: Relative risks concerning in-hospital mortality and one-year mortality between patients admitted on different weekdays in ischemic stroke subset.

| In-hospital mortality | | | |  |  |  |  |  |
| --- | --- | --- | --- | --- | --- | --- | --- | --- |
|  |  | Reference Day | | | | | | |
|  | OR  (95% CI) | Sunday | Monday | Tuesday | Wednesday | Thursday | Friday | Saturday |
|  | Sunday | 1 | 1.000 (0.946~1.057) | 0.972 (0.919~1.028) | 1.013 (0.957~1.072) | 0.997 (0.942~1.055) | 0.959 (0.907~1.014) | 0.994 (0.938~1.053) |
|  | Monday |  | 1 | 0.972 (0.923~1.024) | 1.013 (0.961~1.068) | 0.997 (0.946~1.051) | 0.959 (0.910~1.010) | 0.994 (0.942~1.049) |
|  | Tuesday |  |  | 1 | 1.042 (0.988~1.100) | 1.026 (0.972~1.082) | 0.987 (0.936~1.041) | 1.023 (0.969~1.080) |
|  | Wednesday |  |  |  | 1 | 0.984 (0.932~1.039) | 0.947 (0.897~0.999) | 0.981 (0.928~1.037) |
|  | Thursday |  |  |  |  | 1 | 0.962 (0.912~1.015) | 0.997 (0.944~1.054) |
|  | Friday |  |  |  |  |  | 1 | 1.036 (0.982~1.094) |
|  | Saturday |  |  |  |  |  |  | 1 |

| One-year mortality | | | |  |  |  |  |  |
| --- | --- | --- | --- | --- | --- | --- | --- | --- |
|  |  | Reference Day | | | | | | |
|  | OR  (95% CI) | Sunday | Monday | Tuesday | Wednesday | Thursday | Friday | Saturday |
|  | Sunday | 1 | 1.019 (0.987~1.052) | 0.984 (0.953~1.017) | 0.980 (0.949~1.012) | 0.997 (0.965~1.030) | 0.985 (0.953~1.017) | 1.010 (0.977~1.044) |
|  | Monday |  | 1 | 0.966 (0.938~0.995) | 0.962 (0.933~0.991) | 0.978 (0.949~1.008) | 0.966 (0.938~0.996) | 0.991 (0.961~1.022) |
|  | Tuesday |  |  | 1 | 0.995 (0.965~1.027) | 1.012 (0.982~1.044) | 1.000 (0.970~1.032) | 1.026 (0.994~1.058) |
|  | Wednesday |  |  |  | 1 | 1.017 (0.986~1.049) | 1.005 (0.974~1.036) | 1.030 (0.998~1.063) |
|  | Thursday |  |  |  |  | 1 | 0.988 (0.958~1.019) | 1.013 (0.982~1.046) |
|  | Friday |  |  |  |  |  | 1 | 1.025 (0.994~1.058) |
|  | Saturday |  |  |  |  |  |  | 1 |

Abbreviations: CI, confidence interval; OR, odds ratio.
