# Supplementary material for: Mortality of major cardiovascular emergencies among patients admitted to hospitals on weekends as compared with weekdays in Taiwan
Source: BMC Health Serv Res. 2021 May 29;21:528. doi: 10.1186/s12913-021-06553-7 (PMC8164812; doi:10.1186/s12913-021-06553-7)
Supplement: Supplementary file 8 — Additional file 8 Table S8. Relative risks concerning in-hospital mortality and one-year mortality between patients admitted on different weekdays in pulmonary embolism subset. [file 12913_2021_6553_MOESM8_ESM.docx]

Supplementary Table 8: Relative risks concerning in-hospital mortality and one-year mortality between patients admitted on different weekdays in pulmonary embolism subset.

| In-hospital mortality | | | |  |  |  |  |  |
| --- | --- | --- | --- | --- | --- | --- | --- | --- |
|  |  | Reference Day | | | | | | |
|  | OR  (95% CI) | Sunday | Monday | Tuesday | Wednesday | Thursday | Friday | Saturday |
|  | Sunday | 1 | 1.084 (0.897~1.309) | 1.021 (0.844~1.235) | 1.102 (0.908~1.339) | 1.037 (0.855~1.258) | 0.970 (0.800~1.176) | 1.043 (0.850~1.279) |
|  | Monday |  | 1 | 0.942 (0.799~1.111) | 1.017 (0.858~1.205) | 0.957 (0.809~1.132) | 0.895 (0.756~1.058) | 0.962 (0.803~1.153) |
|  | Tuesday |  |  | 1 | 1.080 (0.910~1.281) | 1.016 (0.857~1.204) | 0.950 (0.802~1.125) | 1.021 (0.851~1.226) |
|  | Wednesday |  |  |  | 1 | 0.941 (0.790~1.120) | 0.880 (0.739~1.047) | 0.946 (0.785~1.140) |
|  | Thursday |  |  |  |  | 1 | 0.935 (0.787~1.111) | 1.005 (0.835~1.210) |
|  | Friday |  |  |  |  |  | 1 | 1.075 (0.894~1.294) |
|  | Saturday |  |  |  |  |  |  | 1 |

| One-year mortality | | | |  |  |  |  |  |
| --- | --- | --- | --- | --- | --- | --- | --- | --- |
|  |  | Reference Day | | | | | | |
|  | OR  (95% CI) | Sunday | Monday | Tuesday | Wednesday | Thursday | Friday | Saturday |
|  | Sunday | 1 | 1.105 (0.951~1.284) | 1.025 (0.881~1.193) | 1.068 (0.916~1.246) | 1.041 (0.893~1.213) | 0.993 (0.851~1.158) | 1.041 (0.886~1.223) |
|  | Monday |  | 1 | 0.927 (0.813~1.058) | 0.967 (0.845~1.105) | 0.942 (0.824~1.076) | 0.898 (0.785~1.027) | 0.942 (0.816~1.087) |
|  | Tuesday |  |  | 1 | 1.042 (0.910~1.194) | 1.015 (0.887~1.163) | 0.968 (0.845~1.110) | 1.016 (0.878~1.174) |
|  | Wednesday |  |  |  | 1 | 0.974 (0.849~1.118) | 0.929 (0.809~1.067) | 0.974 (0.841~1.128) |
|  | Thursday |  |  |  |  | 1 | 0.954 (0.831~1.095) | 1.000 (0.864~1.158) |
|  | Friday |  |  |  |  |  | 1 | 1.049 (0.905~1.215) |
|  | Saturday |  |  |  |  |  |  | 1 |

Abbreviations: CI, confidence interval; OR, odds ratio.
